# Supplementary material for: Apathy in Mild Behavioural Impairment: Associations with Cortical Thickness and Grey Matter Volume
Source: medRxiv. 2026 Feb 27:2026.02.25.26347107. Preprint. [Version 1] doi: 10.64898/2026.02.25.26347107 (PMC12970364; doi:10.64898/2026.02.25.26347107)
Supplement: 1 [file NIHPP2026.02.25.26347107V1-supplement-1.pdf]

medRxiv preprint doi: https://doi.org/10.64898/2026.02.25.26347107; this version posted February 27, 2026. The copyright holder for this preprint (which was not certified by peer review) is the author/funder, who has granted medRxiv a license to display the preprint in perpetuity.

It is made available under a CC-BY 4.0 International license .

Supplementary Table 1 Mean cortical thickness and grey matter volume *Z*-score differences between no-NPS and MBI-apathy groups

	No-NPS (n=387)	MBI-apathy (n=59)	*p*-value
**AD thickness meta-ROI**			
Mean (SD)	-0.28 (0.92)	-0.90 (1.25)	**<0.001[a]**
Median [Min, Max]	-0.19 [-3.70, 1.19]	-0.63 [-3.70, 1.19]	
**ACC thickness**			
Mean (SD)	-0.11 (0.88)	-0.09 (1.17)	0.856[a]
Median [Min, Max]	-0.08 [-2.39, 1.86]	0.02 [-2.39, 1.86]	
**OFC thickness**			
Mean (SD)	-0.36 (0.96)	-0.67 (1.28)	**0.025[a]**
Median [Min, Max]	-0.38 [-3.77, 1.46]	-0.57 [-3.77, 1.46]	
**vlPRF thickness**			
Mean (SD)	-0.28 (0.85)	-0.49 (1.05)	0.088[a]
Median [Min, Max]	-0.27 [-2.40, 1.41]	-0.45 [-2.40, 1.41]	
**dlPFC thickness**			
Mean (SD)	-0.26 (0.92)	-0.72 (1.19)	**0.001[a]**
Median [Min, Max]	-0.23 [-3.29, 1.33]	-0.67 [3.29, 1.33]	
**AD volume meta-ROI**			
Mean (SD)	-0.11 (0.62)	-0.79 (0.81)	**<0.001[a]**
Median [Min, Max]	-0.04 [-2.07, 1.01]	-0.83 [-2.07, 0.80]	
**VS volume**			
Mean (SD)	-0.03 (0.82)	-0.33 (0.97)	**0.012[a]**
Median [Min, Max]	-0.01 [-1.76, 1.67]	-0.25 [-1.76, 1.67]	
**ACC volume**			
Mean (SD)	0.06 (0.65)	-0.06 (0.67)	0.216[a]
Median [Min, Max]	0.05 [-1.47, 1.35]	0.03 [-1.47, 1.35]	
**OFC volume**			
Mean (SD)	0.02 (0.80)	-0.47 (0.89)	**<0.001[a]**
Median [Min, Max]	0.04 [-1.83, 1.55]	-0.57 [-1.83, 1.55]	
**vlPRF volume**			
Mean (SD)	-0.09 (0.66)	-0.21 (0.62)	0.203[a]
Median [Min, Max]	-0.10 [-1.46, 1.32]	-0.20 [-1.46, 1.32]	
**dlPFC volume**			
Mean (SD)	-0.07 (0.69)	-0.45 (0.74)	**<0.001[a]**
Median [Min, Max]	-0.01 [-1.94, 1.17]	-0.48 [-1.94, 0.89]	

NPS = Neuropsychiatric Symptoms; MBI = Mild Behavioural Impairment; VS = Ventral striatum; ACC = Anterior cingulate cortex; OFC = Orbitofrontal cortex; vlPFC = Ventrolateral prefrontal cortex; dlPFC = Dorsolateral prefrontal cortex; ROI = Region of interest; SD = Standard deviation.

Significant *p*-values are shown in bold.

[a]Independent samples *t*-tests.
